# Identifying False Human Papillomavirus (HPV) Vaccine Information and Corresponding Risk Perceptions From Twitter: Advanced Predictive Models

**DOI:** 10.2196/30451

**Published:** 2021-09-09

**Authors:** Tre Tomaszewski, Alex Morales, Ismini Lourentzou, Rachel Caskey, Bing Liu, Alan Schwartz, Jessie Chin

**Affiliations:** 1 School of Information Sciences University of Illinois at Urbana-Champaign Champaign, IL United States; 2 Department of Computer Science University of Illinois at Urbana-Champaign Urbana, IL United States; 3 Department of Computer Science Virginia Polytechnic Institute and State University Blacksburg, VA United States; 4 College of Medicine University of Illinois at Chicago Chicago, IL United States; 5 Department of Computer Science University of Illinois at Chicago Chicago, IL United States; 6 Department of Medical Education University of Illinois at Chicago Chicago, IL United States; 7 Cancer Center at Illinois University of Illinois at Urbana-Champaign Urbana, IL United States

**Keywords:** misinformation, disinformation, social media, HPV, human papillomavirus vaccination, vaccination, causality mining, cause, effect, risk perceptions, vaccine, perception, risk, Twitter, machine learning, natural language processing, cervical cancer

## Abstract

**Background:**

The vaccination uptake rates of the human papillomavirus (HPV) vaccine remain low despite the fact that the effectiveness of HPV vaccines has been established for more than a decade. Vaccine hesitancy is in part due to false information about HPV vaccines on social media. Combating false HPV vaccine information is a reasonable step to addressing vaccine hesitancy.

**Objective:**

Given the substantial harm of false HPV vaccine information, there is an urgent need to identify false social media messages before it goes viral. The goal of the study is to develop a systematic and generalizable approach to identifying false HPV vaccine information on social media.

**Methods:**

This study used machine learning and natural language processing to develop a series of classification models and causality mining methods to identify and examine true and false HPV vaccine–related information on Twitter.

**Results:**

We found that the convolutional neural network model outperformed all other models in identifying tweets containing false HPV vaccine–related information (*F* score=91.95). We also developed completely unsupervised causality mining models to identify HPV vaccine candidate effects for capturing risk perceptions of HPV vaccines. Furthermore, we found that false information contained mostly loss-framed messages focusing on the potential risk of vaccines covering a variety of topics using more diverse vocabulary, while true information contained both gain- and loss-framed messages focusing on the effectiveness of vaccines covering fewer topics using relatively limited vocabulary.

**Conclusions:**

Our research demonstrated the feasibility and effectiveness of using predictive models to identify false HPV vaccine information and its risk perceptions on social media.

## Introduction

About 13,000 women are newly diagnosed with invasive cervical cancer and over 4000 women die from it every year [1]. Cervical cancer is caused by certain types of human papillomavirus (HPV) [2,3]. HPV is the most common sexually transmitted infection in the United States with an estimated 6.2 million new infections every year among persons 14 to 44 years of age [4-6]. In addition to cervical cancer, HPV is the causal mediator in multiple head and neck cancers, genital cancers, and anal cancers [7-9]. The overall burden of HPV-associated cancers has been increasing in the United States [9]. Prevention of HPV is more challenging than most sexually transmitted infections as condoms do not provide complete protection against infection [10]. Hence, prevention through vaccination is critical in decreasing the burden of cancer due to this ubiquitous infection.

The HPV vaccine is universally recommended for all adolescents [10]. Despite the exceptional efficacy (up to 90% protection) in preventing precancerous lesions caused by the targeted HPV types [11-13], only 56.8% of 13 to 17-year-old females and 51.8% of 13 to 17-year-old males in the United States have completed the HPV vaccine series [14]. There are many known barriers to HPV vaccination, including misconceptions about the side effects and adverse events from HPV vaccines, misbeliefs around the need for vaccines, inconsistent advice received from health care givers, costs to complete the vaccination, limited access to clinics, and violations to cultural beliefs [15-21]. Among these barriers, the bias in risk perceptions has not only been associated with low intention of vaccination [22-25] but also with the actual vaccination behavior [16,22,26-30]. The National Immunization Survey revealed the top 3 parental concerns of HPV vaccines to be a lack of knowledge, low perceived usefulness of vaccine (low perceived risk of HPV infection), and high perceived risks of side effects and safety concerns [31], underscoring the importance of risk perceptions in HPV vaccination decisions.

Social media has become an important information source for people to exchange vaccine-related opinions and form their attitudes toward vaccines [32-38]. Its impact is striking, especially for Twitter vaccine information, as HPV vaccine–related opinions on Twitter have been associated with actual vaccine acceptance and coverage [39]. Existing research has investigated the emerging themes and public attitudes toward the pro- and antivaccine online discussions about HPV vaccines [19,24,40-44]. Although multiple false conspiracies and myths around HPV vaccines have been identified, no research has used an automatic computational approach to extract the causal cues of the main vaccination arguments used in circulation of HPV vaccine misinformation. Research has shown that none of content quality, scientific robustness, or the veracity of the information has been found to be indicative of the spread of information, while false or unverified information sometimes becomes more viral than true information [45,46]. As attention to the propagation of false information on social media has surged [45,47-50], an automatic, systematic, and generalizable approach to detect socially endorsed false health information remains understudied. The threats of false information are critical because the reliance (ie, perceived accuracy) on false information can be amplified with each exposure to it and further magnified through social networks [51-53]. People can be especially victimized by the proliferation of user-generated false health information given their lack of health literacy, incompetence in credibility judgments, and the mixed quality of health news despite the sources cited [54-56]. Hence, detecting false health information before it propagates is an important step toward minimizing the threats of false information [57].

Several works have targeted health misinformation [58,59]**,** with most studies using descriptive approaches to study known health misinformation and performing analysis to uncover the common misbeliefs, demographic and geographic patterns, and social media user behaviors [25,60,61]. A few studies have implemented computational models to identify health misinformation from other social medial platforms (such as YouTube and Instagram); however, none of them have attempted to identify health misinformation from short and sometimes incomplete text information, such as tweets [62,63]. In contrast with other related work, we combined a classification model for identifying false HPV vaccine information with unsupervised causality mining to extract the risk perceptions considered to be the attributable causes of HPV antivaccine health concerns based on the content expressed in Twitter messages. To this end, we conducted an infodemiology study to use natural language processing and machine learning methods, such as classification, clustering, dependency parsing, and phrase mining, to identify those false HPV vaccination arguments that frequently appear in social media. Our methodological analysis can be applied to other domains, such as COVID-19 vaccination, food safety, and politics, to extract insightful information regarding the differences and similarities between truthful and misleading claims shared online.

## Methods

**Figure 1:**
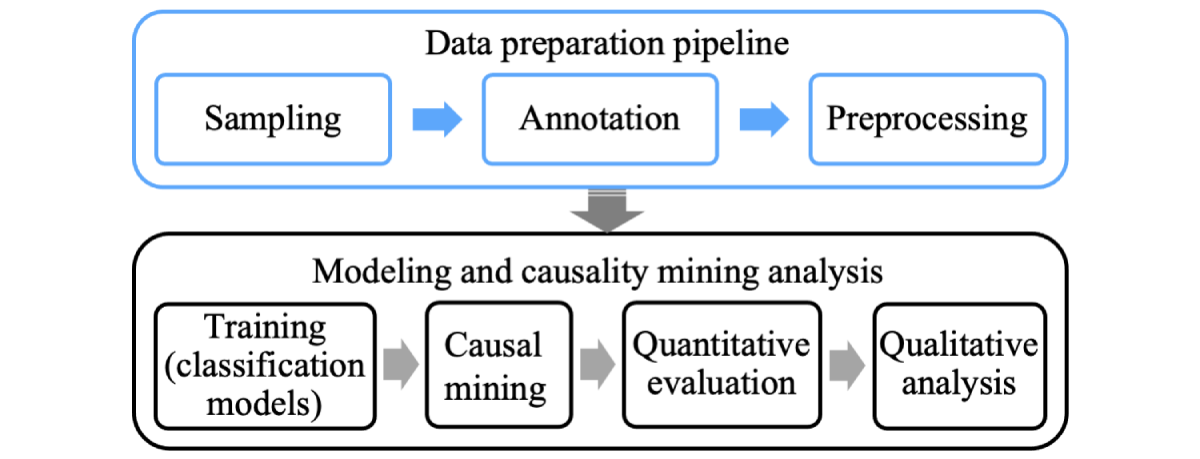
Causality mining data collection and modeling pipeline.

We collected a corpus related to HPV vaccines with tweets published from December 2013 until December 2017. We used the formerly known Crimson Hexagon's (now Brandwatch) social media analytics application programming interface and a list of HPV-related search terms, including, but not limited to, “HPV vaccine,” “papillomavirus vaccine,” “cervical cancer vaccine,” “HPV shot,” “cervical cancer shot,” and “Gardasil.” Our modeling pipeline consists of several steps: sampling, annotation and data preprocessing, training, and analysis (see Figure 1). The data preprocessing stage includes rule-based lexical normalization and unsupervised pretraining of word embeddings.

First, we randomly sampled 1000 tweets per year and passed them to 2 annotators in 2 rounds. Both annotators received basic training about HPV vaccines (including extensive reading of the verified HPV vaccine-related materials from the National Cancer Institute, Center for Disease Control and Prevention, and American Cancer Society), and 1 had formal educational training in medical sciences. Similar to related work in misinformation detection [64-66], we framed the task as a binary classification, in which each tweet is categorized as true or false information (in which false information includes partial-false or partial-true information). We thus not only asked the annotators to judge the veracity of the content for each tweet but also allowed them to select an additional option as “not applicable” for tweets that did not fall under any of the 2 categories (eg, opinionated text and other nonfactual or irrelevant posts). Tweets labeled as not applicable were filtered out from the annotation pipeline. Any discrepancies of the ratings from the 2 annotators were reconciled through discussion. For the interrater reliability, a Cohen's kappa coefficient (κ) of 0.75, was considered to indicate good agreement on the task [67]. The resulting data set consisted of 5000 labeled and 702,858 unlabeled tweets. Character lengths of the tweets, including all mentions, retweets, and hashtags, ranged from 21 to 826 characters.

To reduce vocabulary size for the lexical normalization steps, words were formatted in lower case and URLs were removed; numerals, and Twitter-specific items, such as user mentions (usernames prefixed by “@”) or retweets, were tagged and mapped to a common special token per category (ie, NUMBER, MENTION, RT, respectively). Selected contractions were then replaced with their canonical forms: for example, “Can't” was replaced with “Cannot,” “You'll” was replaced with “You will,” “&” was replaced with “and,” etc. Additionally, hyphens and forward slashes were replaced with spaces, alphanumeric pairings were processed, instances of 2 or more user mentions were reduced to 2 “MENTION” tokens, hashtag quotes and other types of punctuation were removed, and multiple leading or trailing white spaces were replaced with a single one. This process reduced the length of each tweet, which could range between 18 and 295 characters.

The final vocabulary size based on the training set was 4098 terms (including 1 vocabulary term representing a blank space). Analysis of terms weighted by their frequency odds ratio (ie, the ratio of occurrence in each category) showed certain terms were overrepresented in the true category but appeared infrequently in the false category, for example, words that strongly indicated the effectiveness of HPV vaccines on cancer prevention spread online, such as “prevent,” “protect,” and “effective.” On the other hand, false messages contain terms such as “danger,” “adverse,” and “deadly,” and focus more on the negative causal effects that are used as arguments for vaccination.

## Results

### Classification Model

Word embeddings map discrete word tokens to real-valued vector representations, where semantically similar words have similar vectors and are therefore closer in the embedding space. In general, pretraining of such word embeddings has been found to be beneficial for several natural language processing tasks, allowing for faster model convergence and task performance improvements. Therefore, we trained an unsupervised embedding model, FastText [68], with our full Twitter collection as training data and with the aforementioned preprocessing. Compared to other word representation models, FastText can produce word vectors for out-of-vocabulary words and has been proven to be a strong baseline for short text similarity, with its open-sourced implementation allowing for faster training [69]. More specifically, FastText produced 300-dimensional vector representations for each term in our vocabulary, which was used as the initialization for our model's embedding layer. We also experimented with Wikipedia-pretrained embeddings and without any pretrained embeddings: our experiments showed that the model performed better in terms of accuracy when initialized with HPV-related pretrained word embeddings.

**Figure 2:**
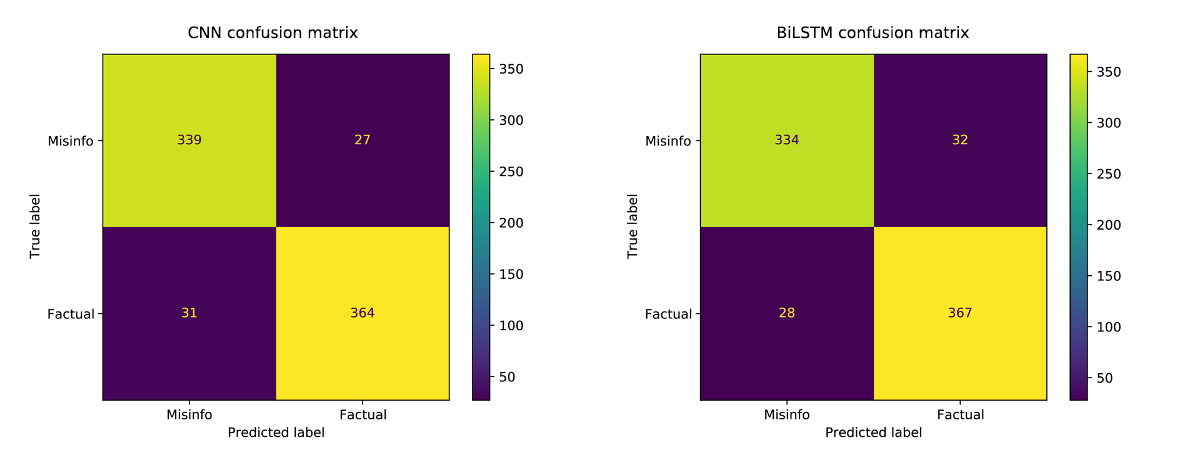
Confusion Matrix for best-performing CNN model. BiLSTM: bidirectional long short-term memory; CNN: convolutional neural network.

**Figure 3:**
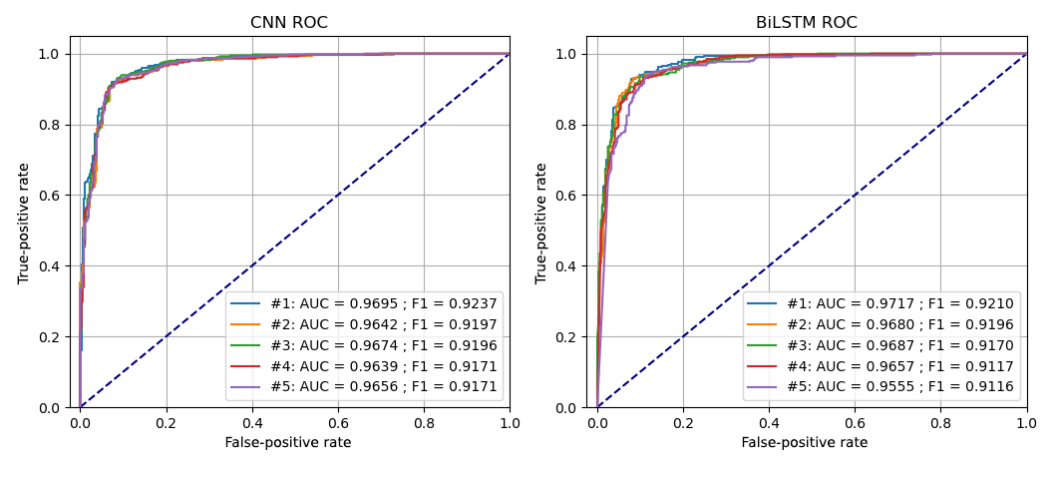
ROC for the best-performing convolutional neural network and bidirectional long short-term memory models. AUC: area under the curve; ROC: receiver operating characteristic.

Finally, we divided the annotated data into 60% training, 20% validation, and 20% testing, keeping the same splits across all models for a fair comparison. Deduplication of tweets with exact matches within each set left 3661 tweets in total (2142 for training, 758 for validation, and 761 for the test set). We experimented with several model architectures, including convolutional neural network (CNN) [70], bidirectional long short-term memory (BiLSTM), and traditional models, including support vector machine and Naive Bayes. We trained with cross-entropy, Adaptive Moment Estimation with a 10^–4^ learning rate, 0.01 decay, and a 32 batch size for the neural models. Hyperparameter tuning was performed using the Tune library [71]. In Table 1, we report the mean and SD of the top-5 performing model variations. Our experimental evaluation showed that CNNs performed better than did the other models (see Figures 2 and 3 for respective confusion matrices and the area under the receiver operating characteristic curve comparisons between neural networks). Of the top-5 best performing models for either of the neural networks, the CNN required less training time than did the BiLSTM. The mean training time per epoch for the CNN was 11.5 ms (SD 1.09, minimum 16, maximum 16, median 12), whereas the mean training time per epoch for the BiLSTM was 51.3 ms (SD 34.07, minimum 14, maximum 88, median 81). Our best-performing CNN model had 256 convolutional filters, including –3 kernels of width (3,4, and 5) and rectified linear unit nonlinearities; a max pooling layer, a fully connected layer of 128 units with rectified linear unit activations and 0.1 dropout, and a final softmax output layer that produced the classification prediction.

**Table 1:** Identifying false human papillomavirus vaccine information: classification model comparison.

Model	Accuracy	Precision	Recall	*F* score
SVM^a^, mean	57.424	57.806	56.721	55.532
Naive Bayes, mean	51.774	52.485	52.301	51.090
CNN^b^, mean (SD)	91.958 (0.269)	91.953 (0.272)	91.946 (0.271)	*91.946 (0.270)* ^c^
BiLSTM^d^, mean (SD)	91.643 (0.432)	91.710 (0.396)	91.574 (0.453)	91.618 (0.438)

^a^SVM: support vector machine.

^b^CNN: convolutional neural network.

^c^Italics indicate the highest *F* score in the table.

^c^BiLSTM: bidirectional long short-term memory.

### Causality Mining

To identify the risk perceptions attributed to the HPV vaccines, we first applied our classifier to a set of 291,037 tweets from which we are able to tag 124,031 as false tweets and 167,006 as true tweets. Using a dictionary of causal terms derived by Kayesh et al [72] for Twitter causality detection, we screened for tweets that contained at least 1 of these terms and kept tweets classified as false information if the classification confidence was at least 0.998, as this maintained high fidelity with our classifier. Thus, a total of 9352 tweets were used for the causal relationship mining process (Table 2). We then used a dependency parser for tweets to tag and merge multi-word expressions [73]. As tweets can have multiple utterances (ie, independent sentences or fragments), we kept the noun phrases that appeared with the causal cue regardless of whether they had a dependency related to the causal cue, which is in contrast to the work by Kayesh et al [72]. A candidate causal phrase is a set of terms pertaining to a tweet that contains a causal cue and precipitates the candidate effect phrase.

**Table 2:** Number of messages after applying several filters.

Model	False	True	Total
No filter, n	124,031	167,006	291,037
+ Confidence threshold, n (%)	72,172 (58.19)	105,166 (62.97)	177,338 (60.93)
+ Contains causal cue, n (%)	3667 (2.96)	5685 (3.40)	9352 (3.21)

We could then compute the pointwise mutual information (PMI) for the causal set *C* = {*c*_1_,...,*c_m_*} and the effect set *E* = {*e*_1_,...,*e_m_*} where the candidate causal phrase, c_i_ and effect phrase, e_j_, are sets that contain terms *w_c_*∈***V***_c_ and *w*_e_∈*V_e_*, respectively. Here, ***V****_c_* is the set of terms, noun phrases, and multi-word expressions derived from candidate causal phrases in the tweets (excluding terms with a minimum frequency of 1 and removing stopwords) and *V_e_* is the vocabulary derived from the candidate effect phrases.

To compute the PMI for terms *w_e_* ∈*e_j_* and *w_c_* ∈*c_i_* we have,



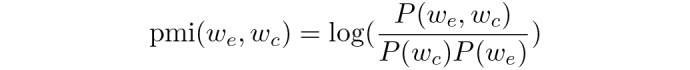



We can apply Laplace smoothing to ensure the probability distributions are nonzero [74] and can compute the normalized pointwise mutual information (NPMI) [75] as follows:



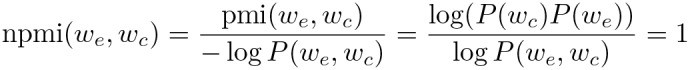



The range of values of NPMI are from –1 to 1, where –1 means the terms never occur together, 0 means they are independent, and 1 is complete co-occurrence.

### Collapsing Candidate Effect Phrases and Ranking Effects

As our model was completely unsupervised and included retweets, tweet messages could become very redundant, but our method could detect many near-duplicate candidate effect phrases. To collapse these phrases, we clustered the terms using semantic similarity derived from embedding representations of the candidate effect phrases. In particular, we used the package HuggingFace [76] to acquire the sum of the last 4 layers of the bidirectional encoder representations from transformers model [77]. To compute the word embedding, we then averaged these word embeddings in the candidate effect phrase to produce the embedding vectors.

Density-based spatial clustering of applications with noise (DBSCAN) [78] was then used to cluster the candidate effect phrases. There were 2 parameters of importance: (1) reachability, which is the max distance between the 2 “points” to be considered in the same cluster; and (2) the minimum number of “points” to be considered clusters. By points here we mean an embedded real-valued vector representation of the effect sets. We set the reachability to 0.1 and the minimum number of points to 1, as we wanted to retrieve only the closest semantically similar words while maintaining meaningful clusters. Note that there are other alternatives to DBSCAN, such as ordering points to identify the clustering structure (OPTICS) [79] and hierarchical density-based spatial clustering of applications with noise (HDBSCAN) [80] for spectral clustering; however, we only needed to reduce the number of effects compared at query time, meaning that DBSCAN was sufficient. We then selected the cluster cores as representatives for each cluster to collapse the effects.

To identify the perceptions associated with different causal words, we formulated this as a retrieval problem. Given a causality-related query *q*, we ranked the associated effects by using the NPMI. To compute the scoring function, we used the following:



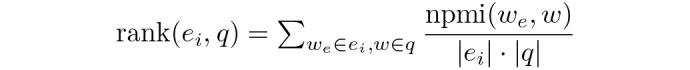



This scoring function computes the average NPMI for all pairs of terms in the query and candidate effect phrase. We can then compute the cumulative NPMI score for a category *C_a_* of effect phrases as follows:



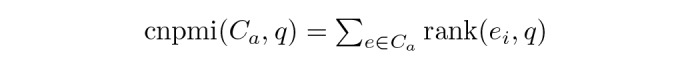



### Causality Mining Results

To validate the candidate causal mining approach, we took a lexicon pertaining to risk perceptions (ie, perceived effects) concerning HPV vaccines. The HPV-Vaccine Risk Lexicon (HPVVR) is a consumer-facing lexicon to capture how laymen describe their risk perceptions about HPV vaccines (including their perceived harms and benefits about HPV vaccines) [81]. The HPVVR was developed in 2 stages. The first stage involved adopting the risk expressions and HPV-vaccine–related consumer-facing vocabulary from the Department of Homeland Security Risk Lexicon, MedlinePlus Consumer Health Topic Vocabulary, and Consumer Health Vocabulary (Unified Medical Language System) [82,83]. The second stage was to extract layman language about the descriptions of risk perceptions based on the user-generated content (from randomly sampled user-generated content from 2013 to 2018, including from Twitter and Facebook) by 2 trained annotators (interrater reliability: Cohen's kappa coefficient (κ)=0.80). The HPVVR covers more than 200 terms or phrases across 29 categories of risk perception-related vocabulary.

This gold standard list of effects, *G*, was then matched with the effect set *E*. In particular, we defined a partial match to be present if some terms in the ground truth effect phrase were matched with some of the candidate effect phrase (ie, *g∩e ≠ Ø*). For example, we mined “prevent throat cancer” from the data, which is a partial match to “prevent cervical cancer” in the HPVVR. There were 2 other kinds of partial match. We defined a match to be proper if the candidate effect phrase was a more specific example of the ground truth effect phrase, *g ∈ G*. For example, we mined “early onset menopause” from the data, which is a proper match to “menopause” in the HPVVR. We considered a reverse match to be present if the candidate effect phrase was a more general form of the ground truth effect phrase, *e ⊆ g*. For example, we mined “fatigue” from the data, which is a reverse match to “extreme fatigue” in the HPVVR. Out of a total of 136 ground-truth effect phrases, we found 55 (40.4%) matches, 78 (57.4%) partial matches, 48 (35.3%) reverse matches, and 103 (75.7%) either partial or proper matches or both. Meanwhile, there were also some candidate effect phrases which were newly discovered effects.

As the causality mining method is a completely bottom-up, unsupervised method, we could automatically mine candidate effects for any set of tweets. In particular, for the predicted false tweets, one of the largest candidate effect clusters contained terms relating to the reactions of different entities, such as “Japan,” “Denmark,” and “college,” on the potential issues with the HPV vaccine, such as “recall,” “lose support,” and “banned.” Another such cluster contained terms relating to infertility misconceptions of the HPV vaccine, such as “premature ovarian failure” and “early menopause on young girls.” Another large candidate effect cluster was about the misconceptions of severe adverse events and complications, such as “sudden death,” “paralysis,” and “stroke.” Note that it is possible that some candidate effect phrases may not be directly related to health effects. Thus, to alleviate this limitation in further analysis, we limited the effects to the terms in the ground truth (ie, *V_e_* = *G*)*.*

## Discussion

### Principal Results

The performance of the CNN and BiLSTM models used in this study showed the feasibility of discerning misinformation from factual information regarding HPV vaccines using the text of tweets. On average, both models predicted either class with high confidence. Although both models performed almost identically in terms of accuracy (and confidence) during testing, the CNN trained much more expediently than did the BiLSTM model, leading to its choice as the preferred model.

**Figure 4:**
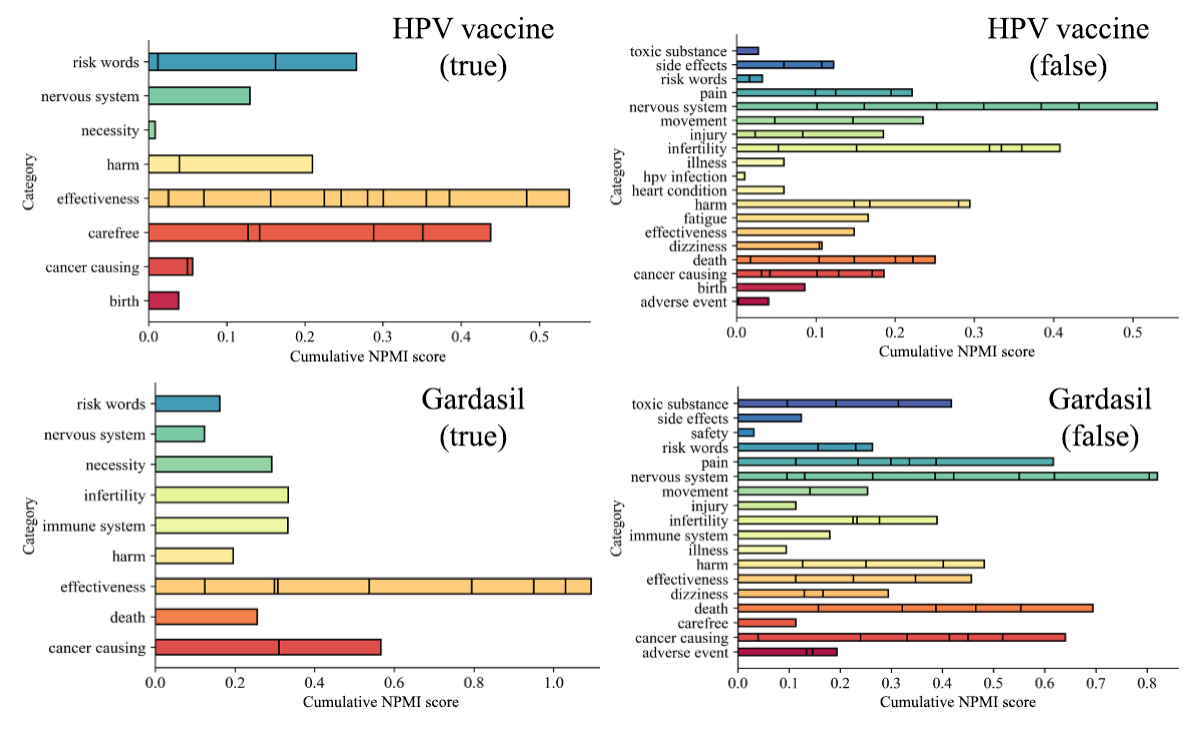
The cumulative NMPI scores when querying for “HPV Vaccine” and “Gardasil”. The sections in the bar width correspond to the NPMI contribution of effect terms for each category. HPV: human papillomavirus; NPMI: normalized pointwise mutual information.

To examine the risk perceptions pertaining to HPV vaccines, we leveraged the false information classifier and the effect ranker. Figure 4 shows the cumulative NPMI scores after our effect ranker querying for both “HPV Vaccine” and “Gardasil” was applied. We could categorize these perceptions around the costs and benefits of HPV vaccines. In general, people discussed the benefits or low risk of harms in the true HPV vaccine tweets and various adverse events in the false HPV vaccine tweets. The main effects associated with the HPV vaccines in the true HPV vaccine tweets were about the prevention of HPV infection–related cancer and the denial of risk of increased unprotected sexual behavior of the vaccinated teens. The main effects associated with the HPV vaccines in the false HPV vaccine tweets were regarding infertility-related conditions (such as ovarian injury), child developmental disorder, death, and toxic ingredients in the HPV vaccines. Following our previous work on the patient-driven HPVVR, the findings from causality mining aided us in identifying the major concerns related to HPV vaccines, whose solutions could then be prioritized.

The results show that false HPV vaccine messages not only span a wide variety of topics in risk perceptions but also involve a more diverse vocabulary to describe these topics compared to the fewer topics and relatively limited terminology found in true messages. This phenomenon of medical-based term frequency and topic diversity within false or misleading messages has also been noted in similar work regarding anti- and provaccine literature [84]. A possible explanation for this discrepancy is that true information requires an evidentiary consensus, thereby restricting terminology and outcomes to a specific selection of topics or phrases used to describe these topics. Misinformation lacks such restrictions to terms or outcomes and tends to use narrative language or mention novel topics to gauge attention [46,85].

We also observed differences in message framing in true and false HPV vaccine messages through causality mining (see Figure 4). True information contained both gain-framed and loss-framed messages, especially those highlighting the effectiveness of the vaccine at preventing HPV-related cancers, the link between HPV infection and cancer, and negating the potential harms of vaccines such as carefree or unprotected sexual behavior (Figure 4). Conversely, false messages were largely loss-framed, focusing on negative outcomes purportedly caused by the vaccine, such as those causing HPV-related cancer or other serious adverse events (infertility, neurological disorder, or death; Figure 4). The use of the risk-indicating causative verb (eg, vaccines “prevent” versus vaccines “harm” or “cause”, etc) might be diagnostic for differentiating the true and false information. Future studies should leverage previous findings on the effectiveness of message framing to examine the impact of misinformation with different framing [86,87].

### Comparison With Prior Work

Health-related misinformation research spans a broad range of disciplines [58,59], with several studies focusing on different medical domains, such as cancer, sexually transmitted disease and infections, influenza, and more recently, COVID-19 [25,60,61]. In vaccine-related domains, several papers have examined vaccine behavior as well as geographic and demographic patterns on the dissemination of antivaccine and misinformation tweets in social media with respect to autism spectrum disorder [60], influenza (flu) vaccines [88], and cancer treatments [89]. Several research endeavors tackle key issues, such as mitigating label scarcity with additional weak social supervision signals, improving intractability with attention mechanisms, and leveraging network and group or user information [65,90-92]. In general, the distinction between vaccine hesitancy identification and vaccination behavior detection is that the former involves an attitude or stance, while the latter is concerned with detecting the action of getting vaccinated [93]. Our study is more similar to the research in vaccine hesitancy but differs in that we focused on extracting causality from tweets through examining risk perceptions; attributable causes of HPV vaccine–related health concerns or expected gain; and using natural language processing, machine learning, and unsupervised causal mining techniques.

We observed that the convolutional models with multiple filter sizes [70,94] worked better than did BiLSTM models for domains with short text, such as tweets. Intuitively, the CNN architecture captures the most common n-grams (of lengths 3, 4, and 5) and therefore is better at discovering discriminative text patterns in short text. Although we tested more sophisticated BiLSTM architectures, overall, the CNN model performed better than did the other model variations and was faster to train. These findings can be useful for social media health–related analysis, in particular with regards to the set of models that practitioners in this domain should explore for social media text classification.

With respect to causality mining, early works use hand-coded, domain-specific knowledge bases [95,96]. A challenge in identifying causal relations is the variety of ways in which it can be observed via various linguistic constructions. A previous study [97] showed that a classifier can determine whether a causal-verb expression, automatically extracted from predefined linguistic patterns of the form <noun phrase–verb–noun phrase> is a causal relationship or not. However, supervised methods require large amounts of manually annotated causes and effects and are thus resource demanding. Recent work has compared unsupervised methods for causal relationship mining including co-occurrence methods, such as point-wise mutual information, and discourse cue-based methods, which are based on information retrieval techniques, to count the number of matches in a cause-effect query [98]. Such comparisons were performed on large-scale document collections, and thus their insights are not applicable to our tasks that, in contrast, have limited amounts of data. Finally, event causality detection in tweets restricts the causal relationship mining to certain events of interest. In “Event causality detection in tweets by context word extension and neural net” [72], the authors propose an approach to encode both the candidate causal phrase and the candidate effect phrase for developing a feed-forward network classifier. Our method is not restricted to certain events. Most importantly, we focus on health-related messages pertaining to HPV vaccines, an approach which can be generalized to other health topics.

### Limitations

One common bottleneck when applying supervised learning methods is the requirement of large amounts of high-quality annotated data for training. Due to the complex nature of the task-at-hand, and the need for extensive manual effort, our data set size might be restrictive in providing insights that can generalize across other domains and data sources. Additionally, due to frequent linguistic variations found in informal user-generated language, closely worded instances might have evaded deduplication. In the future, we hope to address the shortage of available labeled data by incorporating weak supervision methods and denoising mechanisms. Nevertheless, we chose to continue with supervised learning for higher precision, as weak supervision may result in label noise being injected into the false information detection models and thus affecting the subsequent causality mining steps.

Another limitation stems from the misalignment of model confidence and accuracy. In other words, model confidence might not be indicative of model correctness, a problem that is well-known in the machine learning research community [99]. In our experiments, we observed that the BiLSTM model produced high confidence estimates for most false negatives (ie, it misplaced more confidence when predicting factual text), while the CNN model had an equal number of false positives and false negatives for high-confidence examples. Approximately 20% of CNN’s incorrect predictions had low confidence. Overall, the BiLSTM model seems to be overconfident in one direction and could be potentially calibrated better. Further analyses on these high-confidence inaccurate predictions are required to discover interpretable patterns that can identify misinformation subtopics and statements that share strong similarities to factual counterparts.

Finally, we should note that any use of additional metadata requires caution, especially for information that is added by the user, such as user profile characteristics, as well as reported timestamps and social network links, as recent studies show that misinformation spreaders tend to manipulate not only social network structure by forming groups to increase influence [100] but also several types of metadata [101]. In this study, we did not use these types of additional data sources, and thus we can only interpret content-based results and not along any other dimension other than the relationships found in the text.

### Conclusions

The study has demonstrated a systematic, automatic approach to developing computational models for identifying false HPV vaccine–related information and its associated effects on social media. This approach could be generalized to other social media health information and provide insights into estimating the potential effects of a given health topic.

## Abbreviations

BiLSTM: bidirectional long short-term memory
CNN: convolutional neural network
DBSCAN: density-based spatial clustering of applications with noise
HDBSCAN: hierarchical density-based spatial clustering of applications with noise
HPV: human papillomavirus
HPVVR: The HPV-Vaccine Risk Lexicon
NPMI: normalized pointwise mutual information
OPTICS: ordering points to identify the clustering structure
PMI: pointwise mutual information
